# Methodology to calculate forest stand level maximum potential productivity, potential achievable productivity and ecosystem fit

**DOI:** 10.1016/j.mex.2022.101812

**Published:** 2022-08-09

**Authors:** Angela M. Klock, Kristiina A. Vogt, Daniel J. Vogt, John C. Gordon

**Affiliations:** University of Washington, Yale University, USA

**Keywords:** Random forest, Clustering, Binary trees, Reaction norm, Primary productivity, Ecosystem fit

## Abstract

A modified Loomis-William model was originally developed to estimate the theoretical maximum yields of crops. That model was adapted in this paper to measure how much of the theoretical maximum potential productivity (tNpp_tmax_) is reached in any forest due to edaphic and climatic limits to growth, i.e., its “Ecosystem fit” (eFit). The procedure to calculate eFit has not been published except as a concept. Our goal is to describe the methodology in sufficient detail to facilitate its use by the scientific community and forest managers. To calculate eFit you need: 1) to convert all photosynthetically active radiation to a photosynthetic product for each forest plot or stand to calculate its tNpp_tmax_, and 2) use field-collected data of total observed net primary productivity (tNpp_obs_). Theoretical maximum potential tNpp is calculated with a simple light-use efficiency model as the product of the efficiency at which forest canopies absorb solar radiation, the photosynthetic conversion efficiency into biomass, and remotely sensed solar radiation with temperature data extracted to the geographic coordinates for the site. Ecosystem fit represents a forest's realized percent productive capacity and is the ratio of field-collected tNpp (i.e., tNpp_obs_) to the theoretical maximum potential tNpp (i.e., tNpp_tmax_).

• Available indices to assess forest productivity and adaptive capacity to land-use disturbance and climate change are sensitive at the small-to-meso spatio-ecophysiological scales.
• A more holistic index (such as eFit) will provide an informative picture of forest conditions where management practices are undertaken and the ecosystem's capacity to adapt to environmental change.
• A comparison of eFit across similar forests within a climatic zone is an indication of the stressors or constraints that are being imposed locally and that limit tNpp_obs._

## Graphical abstract

**Figure:**
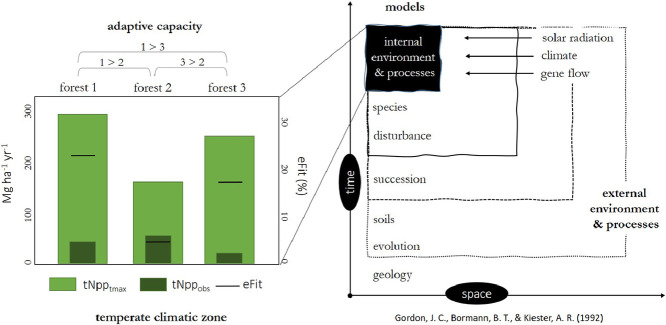

Specifications table

**Table:** 

Subject Area:	Environmental Science
More specific subject area:	Forest ecology and management
Method name:	Calculating forest stand level adaptive capacity to produce biomass
Name and reference of original method:	Loomis, R.S., Williams, W.A. 1963. Maximum crop productivity: An estimate. Crop Sci., 3, 67-72. Gordon, J.C., Farnum, P., Timmis, R., 1983. Theoretical maximum phytomass yields as guides to yield improvement. In: B. Thielges, ed., Proc. 7th N. Am. For. Bio. Work. Univ. Kentucky, Lexington, KY. Gordon, J.C., Bormann, B.T., Kiester, A.R., 1992. The physiology and genetics of ecosystems: A new target or “Forestry contemplates an entangled bank”. Proceedings of the 12th North American Forest Biology Workshop. Sault Ste. Marie, Ontario, Canada. Aug 17-20, 1992. Ontario Ministry of Natural Resources, Ontario Forest Research Institute and Forestry Canada, Ontario Region, pp. 1-14
Resource availability:	N.A.

## Ecosystem Fit rationale to measure adaptive capacity of forests to grow biomass

The “Ecosystem Fit” (eFit) of a forest [8] is the ratio of tNpp_obs_/tNpp_tmax_ (where tNpp_obs_ = total net primary productivity measured at each site and tNpp_tmax_ = theoretical maximum potential total net primary productivity possible when there are no limits to growth), each in units of dry biomass (Mg ha^−1^ yr^−1^) in this paper. It expresses how well a forest is adapted to its site and its potential for growth, i.e., is it possible to grow more biomass than what is measured in the field. Ecosystem Fit [7] directly compares the measured tNpp (or tNpp_obs_ as used in this paper) to growth that is theoretically possible for that site without site-level constraints (i.e., tNpp_tmax_ as used in this paper).

Current measures of tNpp do not allow an assessment of how vulnerable a forest is to climate change since this metric does not identify ranked thresholds of low, medium, or high tNpp by site (*sensu* [9], [21]). In addition, tNpp does not inform how well forests perform in relation to site limiting factors. Thus, comparison of sites requires a productivity metric that defines the maximum potential productivity for each site. For example, forest stand-level comparisons for management strategies could be misleading as an internal reference of gross carbon flux since it could differ unpredictably between sites due to the variability of stand-specific factors affecting productive capacity. Whereas calculating eFit of an ecosystem relative to its current environment can be obtained with the calculation of tNpp_tmax_ as a reference condition to assess the potential of any site to grow biomass [7,8].

Since photosynthesis is the ecophysiological framework where carbon assimilation occurs, it is an indicator to quantify the environment's functional limitations on production [8,14]. Trees convert solar energy into biomass within their genetic and environmental constraints. When all the structural and functional attributes of trees operate at their optimal levels, and the environmental conditions are within the normal range of function, productivity will be maximum for that ecosystem under the conditions present [7].

### Observed tNpp

**Fig. 1:**
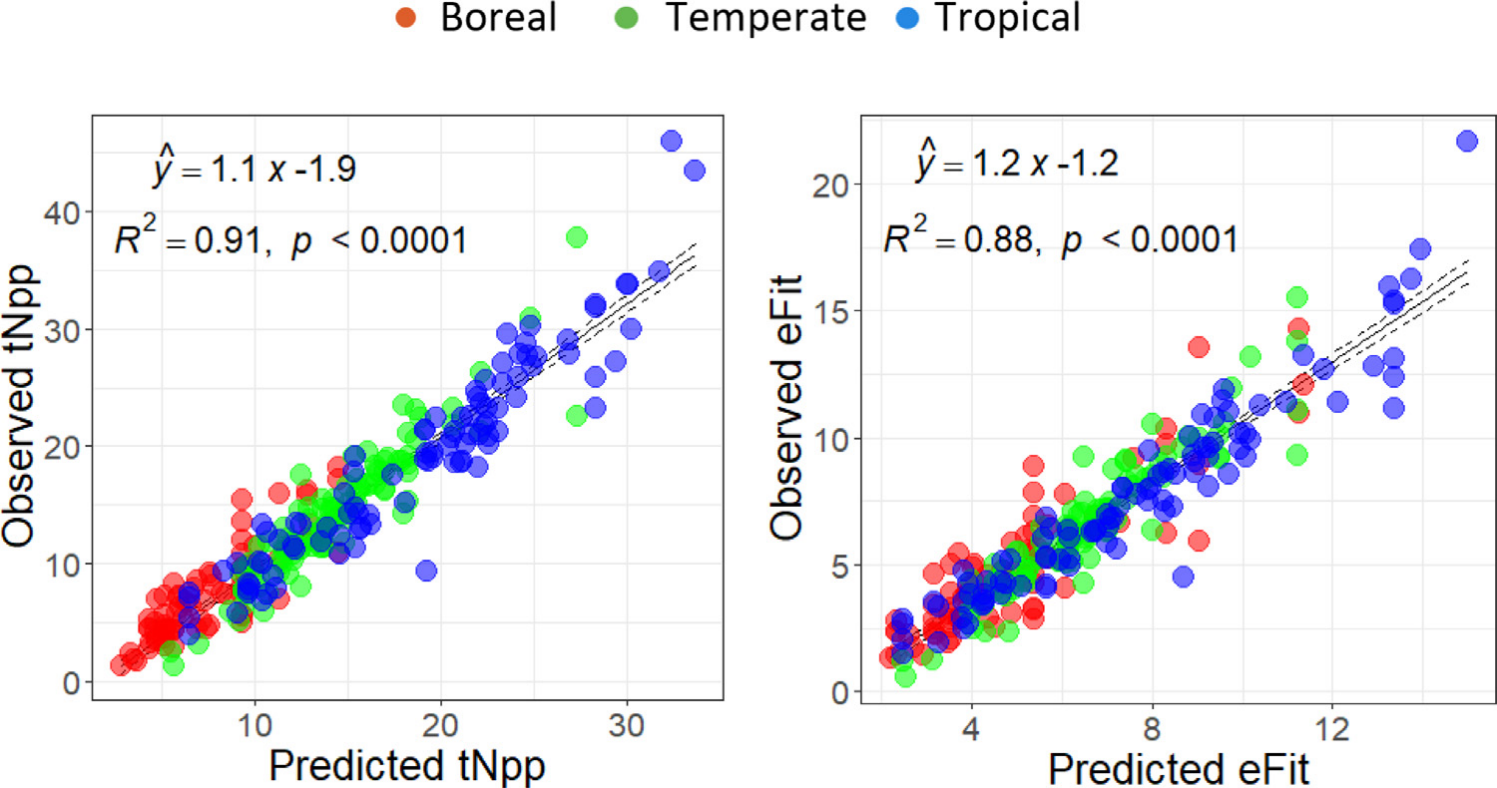
Validation of full Random Forest regression model of field measures of total productivity (tNpp_obs_) and calculated Ecosystem Fit (eFit) for natural forests (*n* = 267). Colors indicate the distribution of the different climatic zones and *R*^*2*^ values are for the full dataset.

The dataset used in this paper consisted of 267 natural forest field sites and 40 forest plantation sites (for a total of *n* = 307) to develop the methodology to calculate plot level maximum potential productivity (tNpp_tmax_), observed or actual productivity (tNpp_obs_), and Ecosystem Fit [9]. The data and variables included in the dataset were comprised of site-level field measures that included above- and below-ground productivity collected from mature boreal, temperate and tropical natural and plantation forests (from [9], Table 1 – variables used in the study database; Fig. 1 – Geographic distribution of sites; and references to individual sites included in our database are in the Supplementary data file - https://doi.org/10.1016/j.ecolind.2022.108973).

## Method details for calculating tNpp_tmax_ and eFit

### Calculating tNpp_tmax_ and eFit

A basic light-use efficiency model was first used to calculate the site-specific theoretical maximum potential net primary productivity without site-level constraints limiting growth. This provided a site-specific maximum tNpp that was then used to calculate Ecosystem Fit. Theoretical potential maximum tNpp (or tNpp_tmax_) is the product of photosynthetically active radiation (PAR) and plant physiological parameters to calculate biomass (*sensu* [4]). This simple efficiency model calculates growth as the product of mean solar radiation available during the growing season [5], the interception efficiency (*Ε_i_*) at which forest canopies absorb solar radiation, and the conversion efficiency (*Ε_c_*) or rate at which absorbed solar radiation is converted into tree biomass. Mathematical equations and model parameter specifications were from Bernacchi et al. [1,2] and the Global Change & Energy Project [6].

The calculation of *Ε_c_* or the conversion efficiency of forest canopies used the average annual atmospheric CO_2_ concentration the year of data collection (1981-2011) for each site as measured by the Mauna Loa Observatory (https://www.sealevel.info/co2.html). Model assumptions were that photorespiration was a constant fraction of photosynthesis, PAR was 45% of total solar radiation, and forest canopies absorbed 90% of incoming PAR during active growth [1]. In addition, we assumed no nutrient or water limitations to growth. Finally, the growing season was calculated as total month-days when mean monthly temperature exceeded zero.

Ecosystem Fit (eFit) is the ratio of measured tNpp (or tNpp_obs_) to the estimated theoretical maximum potential tNpp (or tNpp_tmax_), which is percent eFit (tNpp_obs_/tNpp_tmax_) as shown in Eq. 1:

(1) $$\text{eFit}=\frac{tNpp_{obs}}{tNpp_{tmax}}\times 100$$

where eFit = Ecosystem Fit, tNpp_obs_ = total above- and belowground productivity from field collected tNpp data (Mg ha^−1^ yr^−1^), and tNpp_tmax_ = theoretical maximum potential tNpp (Mg ha^−1^ yr^−1^). Determination of the environmental variables driving eFit percentages were determined independently at each scale by machine learning algorithms with the R packages “randomForest” [3,13], “randomForestSRC,” [18] “caret” [11], and “pdp” [19].

#### Required data acquisition and processing

The methods involved in the development of tNpp_tmax_, eFit, and metrics of forest ecosystem resilience require the following: (1) direct field measures of primary productivity and site-level environmental conditions, (2) global proximal-sensed solar radiation and temperature data, (3) identification of productivity groups using unsupervised clustering, (4) determination of response thresholds, correlations of productivity for each explanatory variable, and the most important environmental variables using binary regression tree analysis, and (5) visualization of response dependence to environmental gradients and low-order interactions that are often critical to understanding ecological processes.

The Random Forest algorithm is not affected by violations of the typical model assumptions of linearity and normality with little/no multicollinearity, and homoscedastic variance. This method has several desirable characteristics: (i) the ability to handle multiple types of data; (ii) the ability to assess interactions and nonlinearity; (iii) the ability to calculate variable importance, and iv) the ability to handle unbalanced data sets [12].

#### Required data

1) Field measures of climatic, environmental, edaphic, and biotic metrics.
2) Total net primary productivity (tNpp) calculated as the sum of field measures of aboveground net primary productivity (aNpp) and belowground net primary productivity (bNpp), and therefore is equivalent to tNpp_obs_ as used in this paper.
3) High spatial resolution average monthly/daily temperature and solar radiation data extracted for the geographic coordinates of each site (buffer zones can be used to calculate a mean for a given area in lieu of point data).

#### Required derived data

1) Determine the length of the growing season, i.e., the number of grow days when the temperature exceeds zero (*G_d_*).
2) Calculate mean temperature for the growing season (*G_t_*).
3) Calculate mean solar radiation for the growing season (*G_srad_*).

#### Model assumptions and parameterization

*R* = molar gas constant (8.314 J mol^−1^ K^−1^)
*K =* specific energy of plant dry biomass (18.2 MJ kg^−1^) [6,16]
*c_a_* = standard or site-/time-level atmospheric concentration of [CO_2_ ppm]
*c_i_* = *c_a_* × 0.7 fraction of intercellular leaf [CO_2_ µmol mol^−1^] [1]
$\varepsilon_{i}$ = interception efficiency at which tree canopies absorb solar radiation (0.90)
β = fraction of absorbed quanta reaching Photosystem II (PSII) (0.45) [1]
$\Gamma^{*}$ = CO_2_ compensation point in the absence of dark respiration (µmol mol^−1^)
  (2) $$\Gamma^{*}=\;\text{exp}\;\left(\frac{c-\Delta H_{a}}{R\;\times \;\left({}^{\circ }C\;+\;273.15\right)}\right)$$

where *c* (kJ/mol) represents a scaling constant and Δ*H_a_* (kJ/mol) represents activation energy, R is the molar gas constant, and the temperature T (° K) measured at each site.

*A* = net rate of CO_2_ uptake after accounting for carboxylation and oxygenation

(3) $$A_{i}=\left(1-\frac{\Gamma^{*}}{c_{i}}\right)\times W_{i}$$

where *Wi* is the rubisco-limited rate of carboxylation (*µ*mol m^−2^ s^−1^)

*W_i_* = *c_i_* / *(*4.5 × *c_i_* + 10.5 × $\Gamma^{*}$) [1,2] $\varepsilon_{c}$ = photosynthetic *conversion* efficiency (rate at which C3 plants convert absorbed solar energy into tissue)

(4) $$\varepsilon_{c}=A\;\times \;\beta \;\times \varepsilon_{i}$$

#### Derive theoretical maximum primary productivity (tNpp_tmax_) and Ecosystem Fit (eFit)

Calculate tNpp_tmax_ as the product of solar radiation during the growing season and plant physiological parameters.

(5) $$\text{tNp}p_{\text{tmax}}=G_{srad}\;\times \varepsilon_{c}\;\times \varepsilon_{i}\times K$$

where *G_srad_* is mean solar radiation available during the growing season and *K* is the inverse specific energy of plant biomass (18.4 MJ kg^−1^).

Photosynthesis is the eco-physiological framework where carbon assimilation occurs. Thus, it is an indicator to quantify the environment's functional limitations on primary productivity [8]. Trees convert solar energy into biomass within their genetic and environmental constraints. If we suppose all structural and functional attributes of trees are operating at their optimal levels, and the environmental conditions are within the tree's normal range of operation, productivity will be maximized for that ecosystem under the conditions present [7]. Based on these constructs, eFit is described as a ratio of measured tNpp_obs_ to the estimated tNpp_tmax_ (tNpp_obs_/tNpp_tmax_) or percent eFit (see Eq. 1).

## Model assumptions and validation of field collected data

This study performed statistical functions in R version 4.0.3 [17], and all analyses were subject to the Shapiro-Wilk tests of normality [22]. Correlations were considered significantly different at *p* < 0.05 and linear regressions were evaluated by behavior of residuals and variance explained. To demonstrate the independence of tNpp and eFit to phenological structure at the global scale and their reliance on stand-level site conditions, comparisons were made with Wilcoxon signed-rank tests for non-parametric and unbalanced data for climatic classifications. Random forest model performance was evaluated by variable importance and which variables (or combinations of variables) and their interactions had the most predictive power. We validated the random forest regression models with visualization of the discrepancies between field observations and predictions of tNpp_obs_ and eFit and calculation of Pearson product-moment correlation coefficient for each (Fig. 1).

**Fig. 2:**
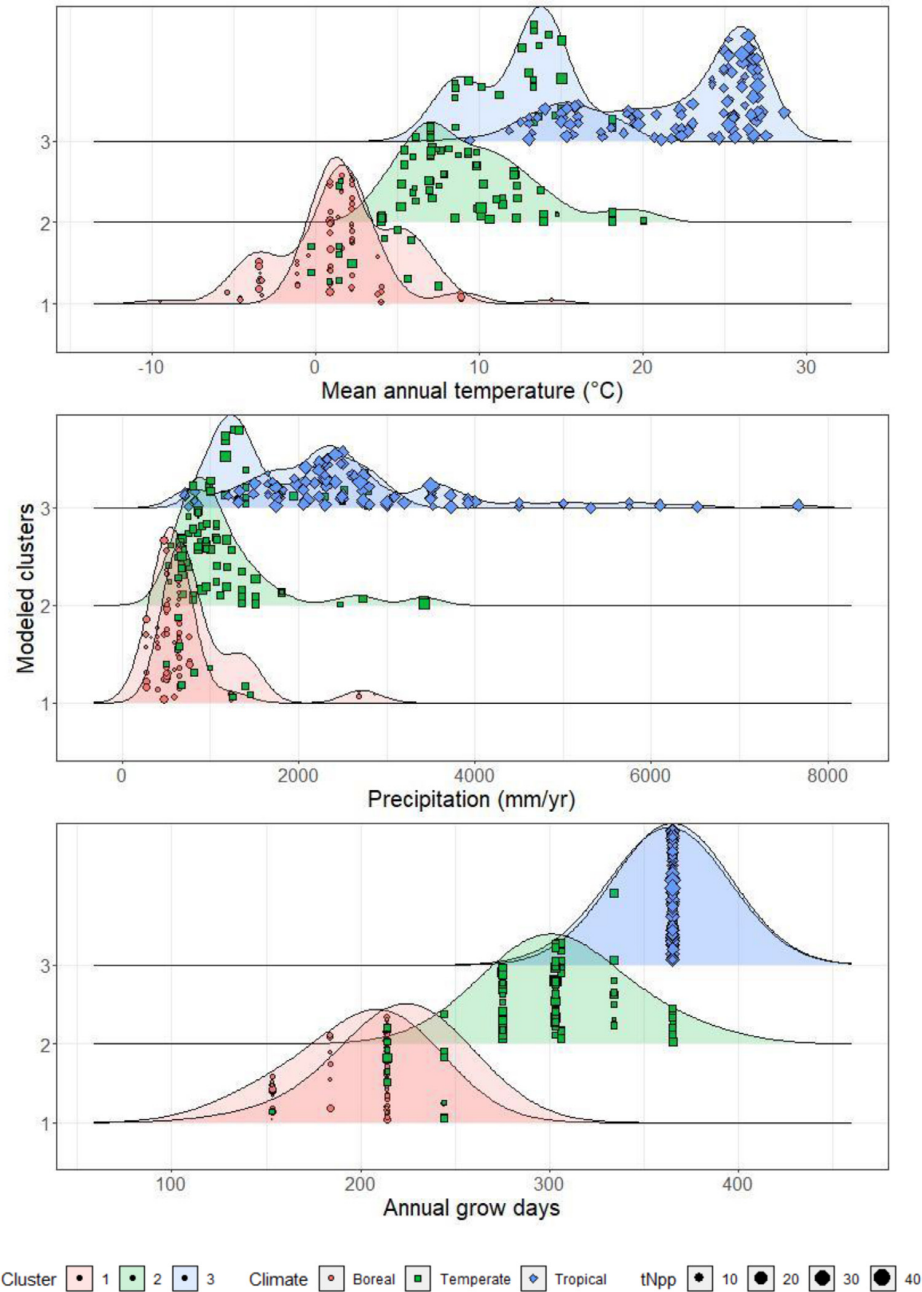
Prediction of climatic classification and density distributions of tNpp_obs_ to the environmental gradient of the three most important variables identified by cluster analysis (represented by the foreground density fill) to the climatic zone designations assigned to the data (represented by the background density fill). Symbols represent the original climatic classification. Color indicates modeled cluster groups and climatic zone. Symbol size represents the decile breaks of tNpp_obs_ for each natural forest ecosystem.

To validate the random forest classification model of the climatic zones we first evaluated the underlying structure of the data by first removing all phenological and climatic membership identifiers. Dissimilarity matrices (distances between all pairs of data points) were calculated for the three most important variables identified by the random forest regression, mean annual temperature, precipitation, and annual grow season. A multivariate random forest model was used to predict the clusters. The data were then aggregated using the partitioning around medoids (PAM) method from the R package “cluster” [20], which repeatedly iterates until the medoid position is stabilized. The medoid of the cluster represents the median of all the attributes included. The optimal number of clusters was selected by evaluating the average silhouette width and the variance explained by the first two principal components. The clusters were visualized with nonlinear dimensional reduction with the R package “Rtsne” [10] utilizing the t-distributed stochastic neighbor embedding technique (t-SNE) [15]. The algorithm first creates probability distributions of the distance relationships between points in their high-dimensional space and then projects them into a reduced 2-dimensional semantic space that retains the proximities of the relationships. This allows identification of misclassifications and density distributions for classes to each environmental variable (Fig. 2).

**Fig. 3:**
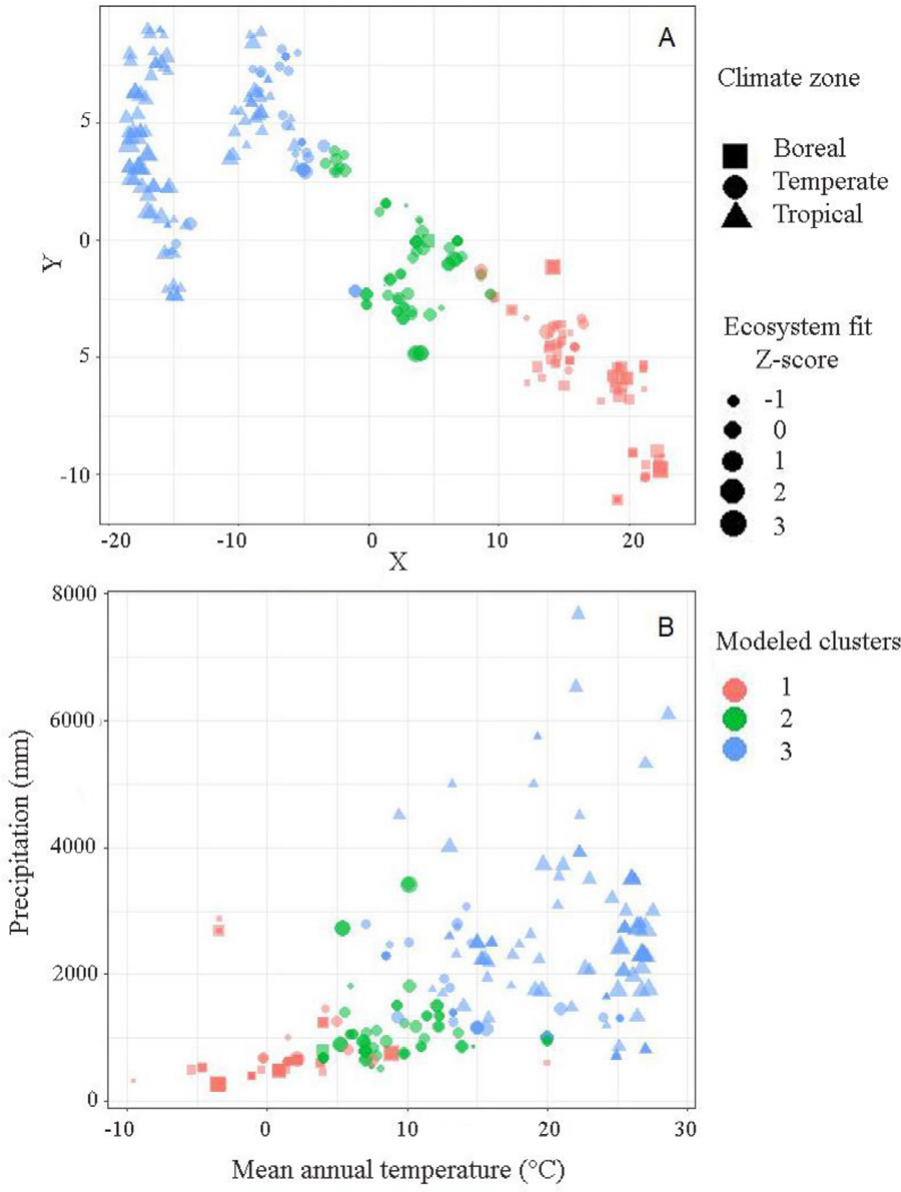
Comparison of model-generated clusters (color) identified by t-SNE and climatic classifications of the data (shapes) of natural forests in (A) semantic similarities in a reduced dimensional space where proximity indicates similarity among forest stands (X and Y axes units have no intrinsic meaning), and (B) distribution in relation to total annual precipitation and mean annual temperature. Symbol size represents the Z-score (standard deviation) of Ecosystem fit.

All class designations were validated by comparing the climatic assignment to model-generated clusters (Fig 3. A) and to the environmental gradients of annual precipitation and mean annual temperature (Fig. 3 B). The Z-score for eFit was added as proportional-sized shapes.

**Fig. 4:**
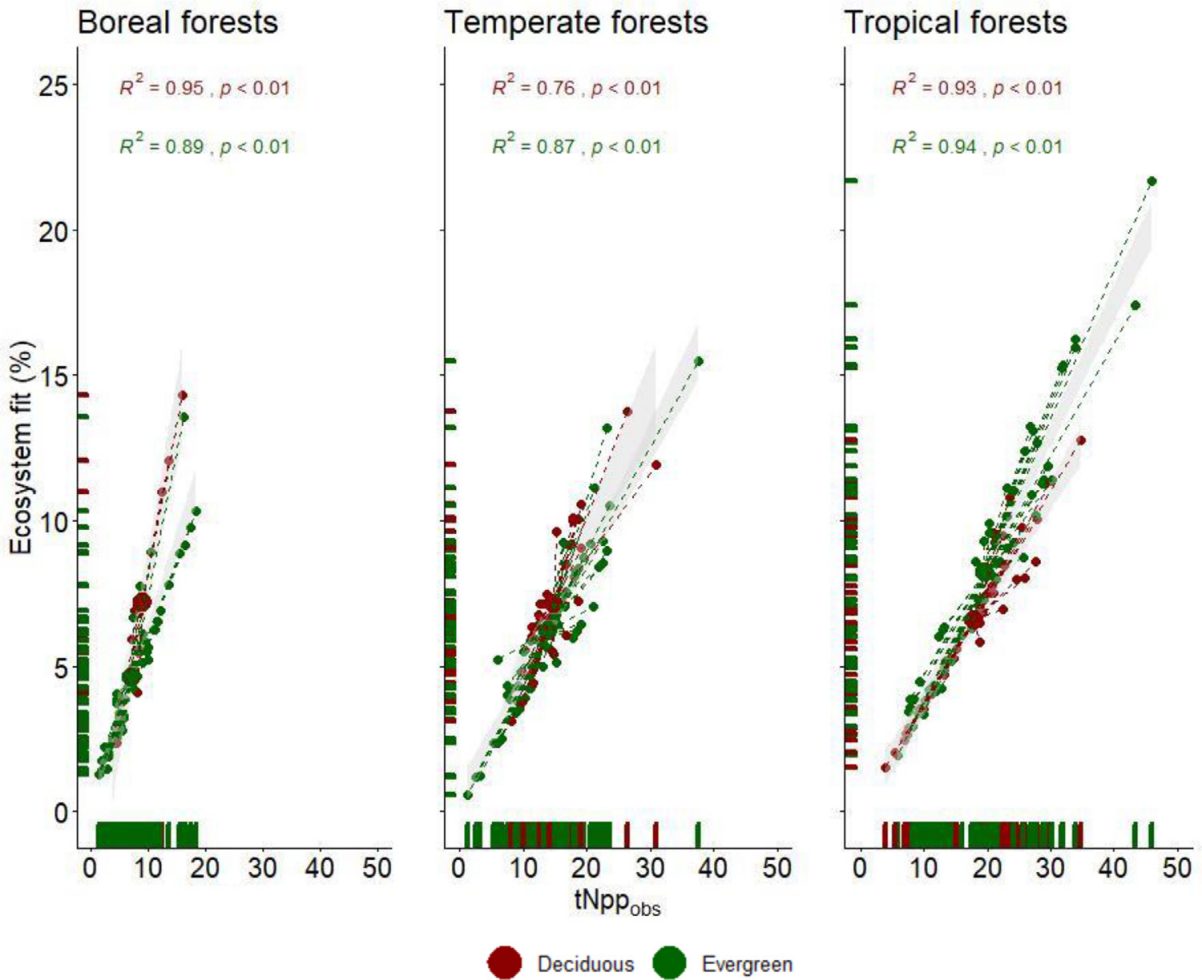
Comparison of the relationship between eFit and tNpp_obs_ in each climatic zone (left to right) and leaf phenology (red = deciduous, green = evergreen). Colored plot rug along the x-axis indicates observations by phenology. The large point is the centroid which represents the mean of all observations and the grey background represents the 95% confidence interval. The statistic is the squared Pearson product-moment correlation coefficient for the corresponding data pairs for deciduous and evergreen natural forests (*n* = 267).

The relationships between tNpp and eFit vary by biome and phenological behavior (Fig. 4). The amount of variance of eFit explained by tNpp was lowest in the temperate forest biome, and especially for deciduous forests where 76% of the variance of eFit was explained by tNpp compared to the evergreen forests (87%). This may be due to the high seasonal variability of the temperate zone compared to the stability of the tropics and strong seasonal forcing and limits of the boreal climate. Thus, if a researcher wants to explain tNpp and eFit in deciduous forests in the temperate climatic zone, other variables need to be explored to improve the strength of the relationship between eFit and tNpp.

**Sample R script:**

##—–SETUP: LOAD REQUIRED PACKAGES————————

require(raster); require(rockchalk); require(dplyr)

##—–LOAD DATA————————

data<-read.csv()

samples <- data.frame(X, Y)

##...create raster stack of mean temperature;

mean.temp <- list.files("",".tif", full.names = TRUE)

meantemp<-stack(mean.temp)

##...rename layers in the raster stack

month <- c("Jan", "Feb", ...)

names(meantemp)<-month

##...extract data from raster layer

meantemp.data <- extract(meantemp, samples)

x<-as.data.frame(meantemp.data)

##...set all negative values to NA

x[x<0]<-NA

##...create raster stack objects for solar radiation;

srad.files <- list.files("”, ".tif", full.names=TRUE)

srad <- stack(srad.files)

##...unique names for layers in the raster stack

month <- c("Jan1", "Feb1", ...)

names(srad)<-month

##...extract data from raster layer

srad.data <- extract(srad, samples);

srad.data<-as.data.frame(srad.data)

##...select solar months based on temperature months

tempsol<-data.frame(x, srad.data)

##...change all NA temps to NA solars

tempsol$Jan1 <- ifelse(tempsol$Jan == "NA", tempsol$Jan1 <-NA, tempsol$Jan1)

...

##...calculate the row means for temps => 0 (growing season)

tempsol$GrowTemp<-rowMeans(tempsol[1:12], na.rm=T)

##...Calculate the row means for solar radiation (*G_srad_*) ###

tempsol$GrowSolar <- rowMeans(tempsol[13:24], na.rm=T)

tempsol$GrowDaysJan<-ifelse(tempsol$Jan1 == "NA", tempsol$GrowDaysJan <- 0, tempsol$GrowDaysJan <- 31)

tempsol$GrowDaysFeb<-ifelse(tempsol$Feb1 == "NA", tempsol$GrowDaysFeb <- 0, tempsol$GrowDaysFeb <- 28)

...

##...column bind variables to new data

dataSoRad<-cbind(data, tempsol[c(25:26,39)])

##...model parameters up front

R <-0.008314 #(kJ mol^^-1^ K^^-1^)

Ca <-400 #CO_2_ concentration (standard or observed)

Ci <-ca*0.7 #280

Beta <-0.45

Alpha <-0.9

Ei <-0.9

K <-18.2 #Inverse specific energy of plant biomass

##...calculate gamma (photorespiration compensation point)

gamma <- exp(19.02 - 37.83 / (R*(GrowTemp + 273.15)))

Wj <- ci / (4.5 * ci + 10.5 * gamma)

##...calculate alpha

A <-(1 – gamma / ci) * Wj

##...calculate photosynthetic efficiency (Ec)

Ec <- A * beta * alpha

##...calculate theoretical maximum NPP (tNpp_tmax_) w/attention to unit conversion

tNpp_tmax_ <- GrowSolar * GrowDays * 0.45 * Ei * Ec * K / units

##...Calculate eco system fit

eFit <- tNpp_obs_ / tNpp_tmax_ # ratio or percent

## Data availability

See supplemental materials [9].

## Declaration of Competing Interests

The authors declare that they have no known competing financial interests or personal relationships that could have appeared to influence the work reported in this paper.

## References

[1] Bernacchi C.J., Pimentel C., Long S.P. (2003). In vivo temperature response functions of parameters required to model RuBP-limited photosynthesis. Plant Cell Environ..

[2] Bernacchi C.J., Singsaas E.L., Pimentel C., Portis A.R., Long S.P. (2001). Improved temperature response functions for models of Rubisco-limited photosynthesis. Plant.

[3] Breiman L. (2001). Random forests. Mach. Learn..

[4] DeLucia E.H., Gomez-Casanovas N., Greenberg J.A., Hudiburg T.W., Kantola I.B., Long S.P., Miller A.D., Ort D.R., Parton W.J. (2014). The theoretical limit to plant productivity. Environ. Sci. Technol..

[5] Fick S.E., Hijmans R.J. (2017). WorldClim 2: new 1-km spatial resolution climate surfaces for global land areas. Int. J. Climatol..

[6] Global Change & Energy Project (2005). An Assessment of Carbon Capture Technology and Research Opportunities. Assessment.

[7] Gordon J.C., Farnum P., Timmis R., Thielges B. (1983). Proc. 7th N. Am. For. Bio. Work. Univ.

[8] Gordon J.C., Bormann B.T., Kiester A.R. (1992). Proceedings of the 12th North American Forest Biology Workshop. Sault Ste. Marie, Ontario, Canada. Aug 17-20, 1992.

[9] Klock A.M., Vogt K.A., Vogt D.J., Gordon J.C., Scullion J.J., Suntana A.S., Mafune K.K., Polyakov A.Y., Gmur S.J., Gómez de la Rosa C. (2022). See the forest not the trees! Ecosystem-based assessment of response, resilience, and scope for growth of global forests. Ecol. Indicators.

[10] J.H. Krijthe, Rtsne: T-Distributed Stochastic Neighbor Embedding using a Barnes-Hut Implementation, (2015) URL: https://github.com/jkrijthe/Rtsne.

[11] M. Kuhn, caret: Classification and Regression Training. R package version 6.0-92. (2022) https://CRAN.R-project.org/package=caret.

[12] Lemon S.C., Roy J., Clark M.A., Friedmann P.D., Rakowski W. (2003). Classification and regression tree analysis in public health: methodological review and comparison with logistic regression. Soc. Behav. Med..

[13] Liaw A., Wiener M. (2002). Classification and Regression by randomForest. R News.

[14] Loomis R.S., Williams W.A. (1963). Maximum crop productivity: an estimate. Crop Sci..

[15] Van Der Maaten L., Hinton G. (2008). Visualizing data using t-SNE. J. Mach. Learn. Res..

[16] Monteith J.L. (1977). Climate and efficiency of crop production in Britain. Philos. Trans. R. Soc. Lond..

[17] R Core Team (2020). R: A language and environment for statistical computing.

[18] Ishwaran H., Kogalur U.B. (2022). Fast Unified Random Forests for Survival, Regression, and Classification (RF-SRC). R package version 3.0.0.

[19] Greenwell B.M. (2017). pdp: An R Package for Constructing Partial Dependence Plots. The R Journal.

[20] Maechler M., Rousseeuw P., Struyf A., Hubert M., Hornik K. (2019). cluster: Cluster Analysis Basics and Extensions. R package version 2.1.2. — See the “Changelog” file (in the package source). /package=cluster..

[21] Vogt D.J., Vogt K.A., Gmur S.J., Scullion J.J., Suntana A.S., Daryanto S., Sigurdardotti R. (2016). Vulnerability of tropical forest ecosystems and forest dependent communities to drought. Environ. Res..

[22] Peat J., Barton B. (2005). Medical Statistics: A guide to data analysis and critical appraisal.

